# Tooth-Ache

**Published:** 1872-04

**Authors:** 


### TOOTH-ACHE.

If a tooth aches, go at once to a good dentist; if there is no decay, it is usually of nervous origin, and if extracted, the pain would go into another tooth; some have uselessly lost several teeth in this way. It is better to use laxative remedies, be out in the open air, and eat half the usual amount. After relief has been obtained, use means to preserve the general health, to prevent the return of the suffering. If the tooth is decayed, it is generally better to have the nerve destroyed, for by this means it can be plugged without pain, and may last for many years.

If the dentist decides that the tooth should be taken out, it is safer and better to let him draw it with your eyes open, and all your senses about you. If you have not courage to encounter the pain, it may be obviated by taking ether, chloroform, or what is known as "laughing gas," as introduced so successfully by Dr. Colton, at the Cooper Institute in New York city; he has taken out many thousands without pain, and without any accident whatever; no loss of life, no fainting, or anything of the kind. Other dentists extract without pain by applying cold to the gum or temples, but the attendant coldness is exceedingly disagreeable. Dr. A. C. Castle's method of benumbing the nerve of the tooth is efficient, safe, successful, and is followed by no annoyance whatever, with the advantage that any intelligent dentist can do the same thing; for he has only to have an assistant to press the middle fingers for a minute against the nerves which lead to the teeth, and thus benumb them, deprive them of all feeling, while the dentist is drawing the tooth. The point of pressure is in the temples, at the little hollow above the cheek bone, and outside of the outer corner of the eye. Life has been often lost by putting various things into the hollow of an aching tooth, creosote, opium, and the like; besides, it affords only a temporary relief, while extraction or nerve killing are permanent. In nearly all cases, premature loss of the teeth could be prevented, if parents would consider it a duty to have their children's teeth most minutely examined, one by one, by a skillful and conscientious dentist, twice a year from five to twenty, and once a year thereafter.

